# Closely related parasitic plants have similar host requirements and related effects on hosts

**DOI:** 10.1002/ece3.7967

**Published:** 2021-07-31

**Authors:** Diethart Matthies

**Affiliations:** ^1^ Plant Ecology Department of Biology Philipps‐Universität Marburg Marburg Germany

**Keywords:** functional groups, legumes, nutrients, parasite–host interactions, root hemiparasites

## Abstract

The performance of root hemiparasites depends strongly on host species identity, but it remains unknown whether there exist general patterns in the quality of species as hosts for hemiparasites and in their sensitivity to parasitism. In a comparative approach, the model root hemiparasites *Rhinanthus minor* and *R. alectorolophus* were grown with 25 host species (grasses, forbs, and legumes) at two nutrient levels. Hosts grown without parasites served as a control. Host species identity strongly influenced parasite biomass and other traits, and both parasites grew better with legumes and grasses than with forbs. The biomass of *R. alectorolophus* was much higher than that of *R. minor* with all host plants and *R. alectorolophus* responded much more strongly to higher nutrient availability than *R. minor*. The performance of the two species of *Rhinanthus* with individual hosts was strongly correlated, and it was also correlated with that of *R. alectorolophus* and the related *Odontites vulgaris* in previous experiments with many of the same hosts, but only weakly with that of the less closely related *Melampyrum arvense*. The negative effect of *R. minor* on host biomass was less strong than that of *R. alectorolophus,* but stronger relative to its own biomass, suggesting that it is more parasitic. The impact of the two parasites on individual hosts did not depend on nutrient level and was correlated. Several legumes and grasses were tolerant of parasitism. While *R. minor* slightly reduced mean overall productivity, *R. alectorolophus* increased it with several species, indicating that the loss of host biomass was more than compensated by that of the parasite. The results show that closely related parasites have similar host requirements and correlated negative effects on individual hosts, but that there are also specific interactions between pairs of parasitic plants and their hosts.

## Graphical abstract

I found that the performance of the two hemiparasites *Rhinanthus minor* and *R. alectorolophus* grown with 25 host species was correlated; that is, plant species that were good hosts for *R. alectorolophus* were also good hosts for *R. minor*. The performance of the *Rhinanthus* species with the individual host species was also correlated with that of *Rhinanthus* in previous experiments with many of the same hosts and with that of the closely related parasites Odontites vulgaris, but not with that of the less closely related *Melampyrum arvense*. The results show that closely related parasites have similar host requirements and have correlated negative effects on individual hosts, but that there are also specific interactions between pairs of parasitic plants and their hosts that will result in different impacts on host communities.

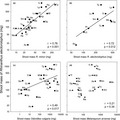

## 1 INTRODUCTION

Root‐hemiparasitic plants have green leaves and are photosynthetically active, but attack the roots of other plants and extract water and solutes from them (Cameron & Phoenix, 2013; Phoenix & Press, 2005). While hemiparasites may use a wide range of plant species as their hosts, different species vary in their quality as hosts and the identity of the host strongly influences the growth of hemiparasites (Calladine et al., 2000; Hautier et al., 2010; Matthies, 2017; Nge et al., 2019; Rowntree et al., 2014; Sandner & Matthies, 2018). The suitability of a species as a host for a hemiparasite depends on the quantity and quality of compounds the parasites obtains from the host (Atsatt, 1983; Govier et al., 1967) and on the strength of resistance it has against the attack by the hemiparasite (Cameron et al., 2006). Because hemiparasites usually have a very reduced root system (Matthies, 2017), the uptake of water and nutrients from the host is thought to be the most important benefit of parasitism, but hemiparasites can also obtain significant amounts of carbon from their hosts (Press et al., 1988; Tennakoon & Pate, 1996; Těšitel, Plavcová et al., 2010; Těšitel, Těšitelová, et al., 2015).

Based on studies with species of the genus *Rhinanthus*, it has been concluded that legumes are particularly good hosts for hemiparasites, followed by grasses, while nonleguminous forbs are less suitable as hosts (Cameron & Phoenix, 2013). However, in a large study with the hemiparasite *Melampyrum arvense* forbs were on average better hosts than grasses (Matthies, 2017). There is also considerable variation in host quality within functional groups (Hautier et al., 2010; Matthies, 2017; Rowntree et al., 2014). For example, in spite of their high N‐content not all legumes are good hosts for hemiparasites (Matthies, 1998; Nge et al., 2019; Radomiljac, 1999). The legume *Anthyllis vulneraria* was found to be a poor host for both *Melampyrum* and *Rhinanthus*, and *Onobrychis viciaefolia* was an unsuitable host for *R. alectorolophus* (Matthies, 2017; Sandner & Matthies, 2018). Moreover, the results of studies on the quality of individual species as hosts for hemiparasites have often been inconsistent. For example, *Anthyllis* was a good host for *Euphrasia* ssp. (Yeo, 1964), and while *Trifolium repens* was a good host for *Rhinanthus angustifolius* (De Hullu, 1984), it was a poor host for two species of *Odontites* (Snogerup, 1982). These inconsistent results could be due to specific interactions between hemiparasite–host pairs, but could also be due to differences in experimental conditions. Because in most studies, a hemiparasite species was grown with only one or a few host species, effects of host species and experimental conditions cannot be separated, which severely restricts the value of comparisons of the relative performance of parasites with the various hosts. It is thus not known whether the performance of different species of hemiparasites with individual host species is correlated.

Hemiparasites often have strong negative effects on the growth of their host plants, because they extract water, nutrients, and carbon from them and may reduce host photosynthesis (Matthies, 1995; Phoenix & Press, 2005). However, there is strong variation in the sensitivity of plant species to parasitism. While growth and reproduction of some species are strongly reduced by a hemiparasite, others are resistant against parasite attack (Cameron et al., 2006), and some species are tolerant of parasitism; that is, they are good hosts and provide strong benefits to the parasites but are not harmed by them (Matthies, 2017; Sandner & Matthies, 2018). However, it is not known whether the resistance or tolerance of an individual host species is parasite‐specific, or whether the response of potential host plants to different species of parasites is similar.

In most experiments, root hemiparasites have reduced overall productivity (Ameloot et al., 2005; Hautier et al., 2010; Matthies, 1995, 1996), indicating that their resource use efficiency is lower than that of their host plants (Matthies, 1995; Těšitel, Těšitelová, et al., 2015). It has therefore been proposed to use hemiparasites to reduce the productivity of grasslands of interest for conservation (Demey et al., 2015; Pywell et al., 2004; Těšitel et al., 2017; Westbury et al., 2006). However, although it has been predicted that the productivity of hemiparasite–host systems will always be lower than that of the communities without the parasite (Hautier et al., 2010), in some studies hemiparasites had no effect on overall productivity or even increased it (Joshi et al., 2000; Sandner & Matthies, 2018).

**FIGURE 1:**
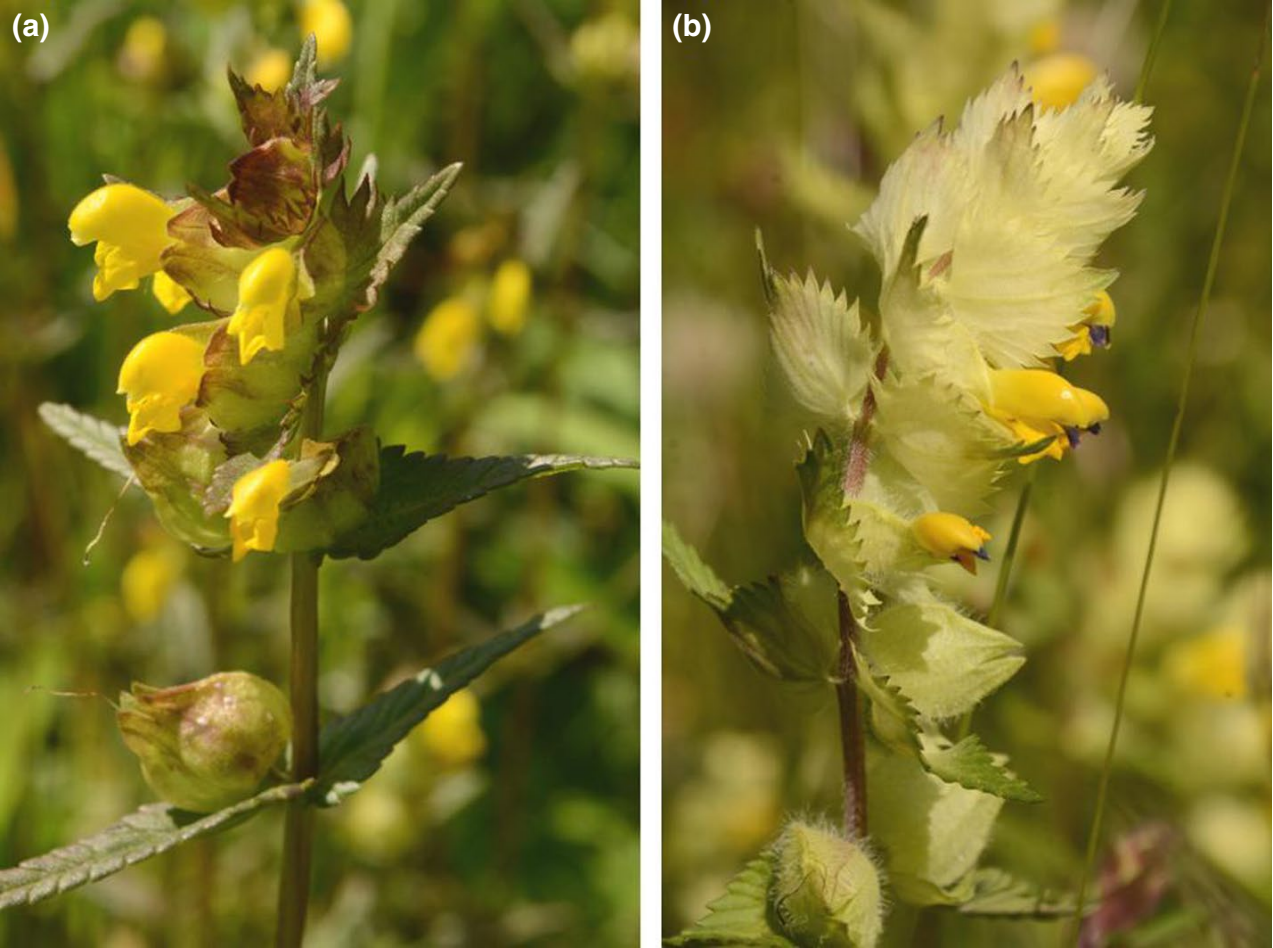
(a) *Rhinanthus minor* and (b) *Rhinanthus alectorolophus*

The aim of the present study was to comparatively investigate the interactions of two closely related hemiparasites, *Rhinanthus alectorolophus* (Scop.) Poll. and *R. minor* L. (Orobanchaceae; Figure 1), with a large number of species. *Rhinanthus* species have been widely used as models for the investigation of hemiparasite–host relationships (Cameron & Phoenix, 2013; Matthies, 1995; Rowntree et al., 2014; Sandner & Matthies, 2018; Seel & Press, 1994; Těšitel, Těšitelová, et al., 2015). The two species of *Rhinanthus* were grown with 25 different host species, many of which had also been used in some previous experiments with hemiparasites. This made it possible to compare the performance of the two parasites with the individual hosts and with that of other hemiparasites grown with the hosts in previous studies. The host species were in addition grown without a parasite to compare the effects of the two parasites on the host species. As hemiparasite–host relations may be influenced by nutrient availability (Korell et al., 2020; Matthies, 2017; Matthies & Egli, 1999; Těšitel, Těšitelová, et al., 2015), all plant combinations were also grown at two levels of nutrients. I asked the following specific questions: (i) Is the performance of the two parasite species grown under the same conditions with individual host species correlated, that is, are plant species that are good hosts for *R. alectorolophus* also good hosts for *R. minor,* and is the performance of the *Rhinanthus* species with individual host species correlated with that of *Rhinanthus* in other experiments and with that of other closely related hemiparasites? (ii) Is the effect of the two parasites on individual host species correlated? (iii) Are the interrelationships between the two hemiparasites and different hosts influenced by nutrient level?

## 2 MATERIALS AND METHODS

### 2.1 Parasites studied

*Rhinanthus minor* L. and *R. alectorolophus* (Scop.) Poll. (Orobanchaceae) are annual root hemiparasites that attack a wide range of host species (Hautier et al., 2010; Rowntree et al., 2014; Sandner & Matthies, 2018), but can also grow without a host (Matthies & Egli, 1999; Seel & Press, 1993), although less vigorously. Seeds of *Rhinanthus* spp. germinate in autumn at low temperatures, but during the winter, the epicotyl is dormant and only the hypocotyl develops (Hartl, 1974; Westbury, 2004). The seedlings emerge above ground in March to April and grow rapidly, and the parasites start flowering in May. The flowers are pollinated by bumblebees (Kwak, 1979), but may also self‐pollinate (Sandner & Matthies, 2017).

*Rhinanthus minor* is widespread throughout Europe and has also been introduced to North America (Westbury, 2004), where it recently has become invasive (Smith & Cox, 2014), while *R. alectorolophus* has a more restricted distribution in Central Europe (Hartl, 1974). Both parasite species are mainly plants of grasslands. While *R. minor* is a plant of nutrient‐poor grasslands, *R. alectorolophus* grows typically in more nutrient‐rich habitats than *R. minor* (Ellenberg et al., 1992) and was formerly also a weed of cereal crops in Europe (Hartl, 1974).

### 2.2 The experiment

The hemiparasites *R. minor* and *R. alectorolophus* were grown with 25 host species at two nutrient levels (Table 1). All the host species occur with the *Rhinanthus* species in their habitats. Seeds of both the parasites and the hosts were obtained from a commercial supplier (Appels Wilde Samen, Darmstadt, Germany). The host species were selected to include species from the functional groups grasses, legumes, and nonleguminous forbs and from nutrient‐poor and moderately nutrient‐rich habitats. Each species was assigned to one of three groups according to its Ellenberg indicator value for nutrients (Ellenberg et al., 1992). Species with *N*‐values of 2–3 were classified as species of low‐nutrient habitats, those with *N*‐values of 4–6 as species of moderately nutrient habitats, and those with *N*‐values of 7–8 as species of high‐nutrient habitats. Species for which no *N*‐values were available were classified as indifferent.

**TABLE 1:** Species used as host plants in the experiment. The indicator value for nutrients (*N*‐value, Ellenberg et al., 1992) indicates the realized ecological niche of a species with respect to nutrient level in Central Europe. Species with *N*‐values of 2–3 were classified as species of low‐nutrient habitats, those with *N*‐values of 4–6 as species of medium‐nutrient habitats, and those with *N*‐values of 7–8 as species of high‐nutrient habitats. Species for which no *N*‐values were available, because their behavior is indifferent to nutrients, were classified as indifferent

Host species	Species code	Family	*N*‐value	Nutrient status of habitat	Functional group
*Hieracium pilosella*	Hp	Asteraceae	2	Low	Forb
*Sanguisorba minor*	Sm	Rosaceae	2	Low	Forb
*Chrysanthemum leucanthemum*	Cl	Asteraceae	3	Low	Forb
*Koeleria pyramidata*	Kp	Poaceae	2	Low	Grass
*Bromus erectus*	Be	Poaceae	3	Low	Grass
*Lotus corniculatus*	Lc	Fabaceae	3	Low	Legume
*Achillea millefolium*	Am	Asteraceae	4	Medium	Forb
*Capsella bursa‐pastoris*	Cb	Brassicaceae	5	Medium	Forb
*Anthoxanthum odoratum*	Ao	Poaceae	x	Indifferent	Grass
*Myosotis arvensis*	Ma	Boraginaceae	6	Medium	Forb
*Papaver rhoeas*	Pr	Papaveraceae	6	Medium	Forb
*Plantago lanceolata*	Pl	Plantaginaceae	x	Indifferent	Forb
*Daucus carota*	Dc	Apiaceae	4	Medium	Forb
*Trisetum flavescens*	Tf	Poaceae	5	Medium	Grass
*Cynosurus cristatus*	Cc	Poaceae	4	Medium	Grass
*Dactylis glomerata*	Dg	Poaceae	6	Medium	Grass
*Medicago lupulina*	Ml	Fabaceae	x	Indifferent	Legume
*Medicago sativa*	Ms	Fabaceae	5	Medium	Legume
*Trifolium pratense*	Tp	Fabaceae	x	Indifferent	Legume
*Taraxacum officinale*	To	Asteraceae	7	High	Forb
*Urtica dioica*	Ud	Urticaceae	8	High	Forb
*Arrhenatherum elatius*	Ae	Poaceae	7	High	Grass
*Lolium perenne*	Lp	Poaceae	7	High	Grass
*Poa annua*	Pa	Poaceae	8	High	Grass
*Trifolium repens*	Tr	Fabaceae	7	High	Legume

Seeds of *R. minor* and *R. alectorolophus* were set up for germination on moist filter paper in Petri dishes in mid‐February and kept at 5°C to break dormancy. At the beginning of May, 10–15 seeds of the host plants were sown into pots of 9 × 9 × 9.5 cm filled with nutrient‐poor commercial soil (TKS Instant, Floragard, Oldenburg, Germany), except for seeds of *Achillea millefolium*, *Poa annua,* and *Lolium perenne*, which were known to germinate faster and were sown one week later. The pots were kept in an unheated glasshouse. After three weeks, the number of seedlings was reduced to three per pot. At the end of May, one seedling of *R. minor* or *R. alectorolophus* was transplanted into the center of a number of pots with each host species. After initial transplanting mortality, c. 10 replicates (means: *R*. *minor* 9.9*, R. alectorolophus:* 10.4*;* range 9–13) per combination of each host and parasite species remained. In addition, ten pots with each host species were left as no‐parasite controls. These pots were then placed on saucers in flower beds in the Botanical Garden of the University of Marburg. In a further ten pots with each host species, the biomass of the hosts was harvested above ground, dried for 48 hr at 80°C, and weighed to obtain a measure of initial host size.

During the first two weeks, the plants were protected against the sun with shading cloth that reduced the light intensity by 45%. Two weeks and four weeks after the planting of the parasites, half of the pots received 40 ml of a 0.3% solution of Wuxal Super (Aglukon, Düsseldorf; N‐P‐K: 8%‐8%‐6%) fertilizer, and six weeks after planting, they received another 40 ml of a 0.4% solution of the fertilizer (high‐nutrient level) to ensure differences in host growth between the two treatments, while the other pots received only water (low‐nutrient level).

After four weeks of growth, the length of the longest leaf of each parasite was measured as an nondestructive estimate of size. Once the parasites started to flower, the date when the first flower opened was recorded for each plant. In the 10th week after planting when the parasites were fruiting, the length of the longest leaf, the height, and total inflorescence length of each parasite were measured. Parasites and hosts were then separately harvested above ground, dried for 48 hr at 80°C, and weighed. Mortality of the parasites was calculated as the proportion of parasites that died after they had survived the first ten days after transplanting. Fifty seeds of each host species were weighed to obtain its mean seed mass, because host seed mass might influence their early growth rate. The relative growth rate (RGR) of the host plants grown without a parasite was calculated as ln(mean biomass at harvest) − ln(mean mass at planting of *Rhinanthus*) divided by the duration of host growth (63 d for *Achillea*, *Lolium* and *Poa*, 70 d for all other hosts).

### 2.3 Statistical analyses

The influence of host species and nutrient level on the mortality of the parasites was analyzed with chi‐square tests. The effects of the two treatments on the biomass and other traits of the two parasites were studied by two‐factor analyses of variance. Because these traits are not independent, *p*‐values were adjusted for the false discovery rate (Benjamini & Hochberg, 1995). To investigate whether the quality of a host species for the two species of *Rhinanthus* was correlated, the relationship between the biomass of the two species with the same hosts was studied by linear regression.

Because many of the hosts used in the current experiment had been used in a previous study of the host relations of the related hemiparasite *Melampyrum arvense* (Matthies, 2017), it could also be studied whether good hosts for *Melampyrum* were good hosts for *Rhinanthus* by correlating the biomass of the *Rhinanthus* species with that of *M. arvense* grown with the same hosts. The performance of *Rhinanthus* with the individual hosts was further related to the results of another study with *R. alectorolophus* (Sandner & Matthies, 2018) and one with the parasite *Odontites vulgaris* (Geppert, 2012).

To analyze the effects of various host traits on the biomass of the parasites, linear mixed models were constructed separately for the two parasite species with host species identity as a random factor and the following fixed factors: nutrient level, nutrient status of the typical habitat of the hosts, mean seed mass of the hosts, mean biomass of the hosts at the start of the experiment, mean final biomass of the hosts grown with or without a parasite, and mean RGR of the hosts. The metric explanatory variables were standardized for the analyses. In a second step, all possible models including the explanatory variables were calculated and ranked by their AICc to obtain the best models. Differences between the performance of the parasites growing with species from different functional groups were then analyzed using Tukey‐adjusted *p*‐values.

To analyze the effect of the size of the host plants growing in the same pot on the biomass of the individual parasites, the mean biomass of *R. minor* and *R. alectorolophus* was related in general linear models to nutrient level, host species identity, their interaction, and host mass. The mean biomass of the parasites was also related to the length of their longest leaf after four weeks of growth to assess the influence of early differences in size on final biomass.

The effects of host species, parasite species, nutrient level, and their interactions on host biomass and total aboveground productivity (host + parasite) per pot were studied by three‐way analyses of variance. To investigate whether damage to a host and benefit to a parasite were correlated, log‐response ratios were calculated for the effect of the parasites on the individual host species as log (mean biomass of a host with a parasite/mean biomass without a parasite) and related to the mean biomass of the parasites achieved with the individual host species.

All analyses were carried out with R 4.0.3 (R Core Team, 2020). Analyses of variance and general linear models were carried out with the package *lm*. *p*‐Values using type III sums of squares were obtained with the ANOVA function of the *car* package (Fox & Weisberg, 2019). *p*‐Values adjusted for the false discovery rate were obtained with the *p.adjust* command. Linear mixed models were carried out with the function *lmer* of package *lme4* (Bates et al., 2015). All possible linear models and their AICc values using a set of explanatory variables were calculated with the *dredge* function of the *MuMIn* package (Barton, 2020). Mean values and Tukey‐adjusted *p*‐values were obtained with the *emmeans* package (Lenth, 2020). Data for biomasses, height, inflorescence length, and seed mass were log‐transformed prior to analysis to obtain normally distributed residuals and homoscedasticity.

## 3 RESULTS

### 3.1 Influence of the host species on parasite traits

There was no evidence that the mortality of the two hemiparasites (*R. minor*: 41%, *R. alectorolophus*: 19%) was influenced by the host species (*R. minor*: χ^2^ = 16.5, *df* = 24, *p* = 0.87; *R. alectorolophus*: χ^2^ = 33.4, *df* = 24, *p* = 0.18) or by the nutrient level (*R. minor*: χ^2^ = 0.51, *df* = 1, *p* = 0.48; *R. alectorolophus*: χ^2^ = 0.81, *df* = 1, *p* = 0.37).

**FIGURE 2:**
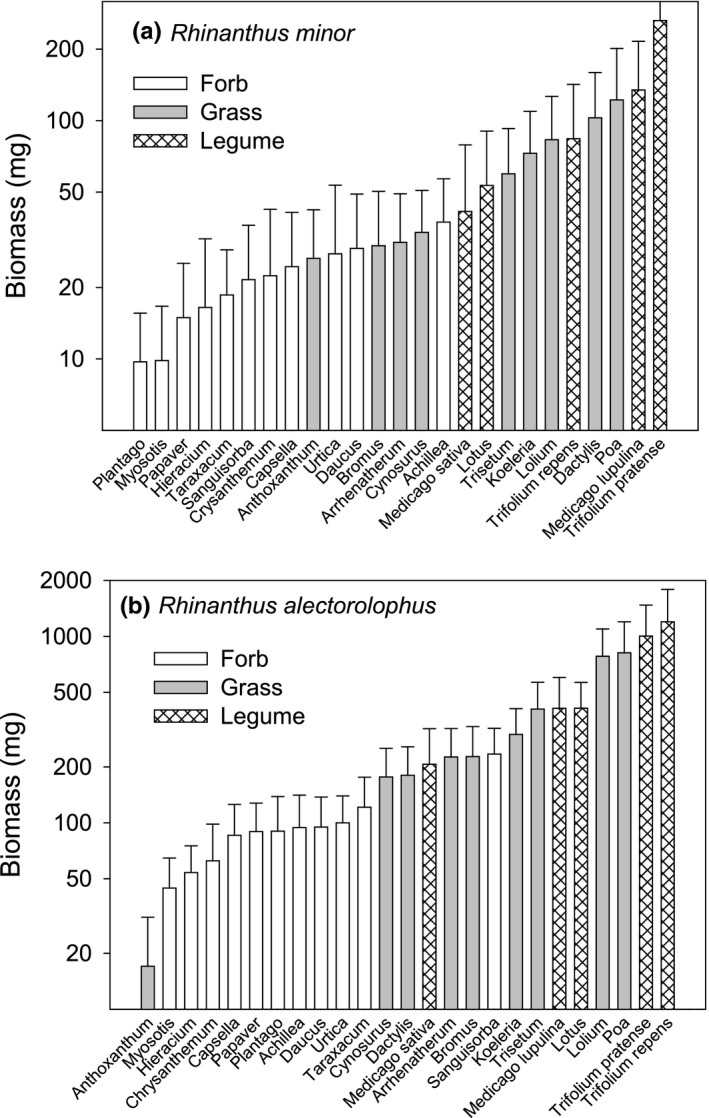
The biomass of the hemiparasites (a) *Rhinanthus minor* and (b) *Rhinanthus alectorolophus* grown with 25 different host species (legumes, grasses, and nonleguminous forbs). The host species are in increasing order of parasite biomass

**FIGURE 3:**
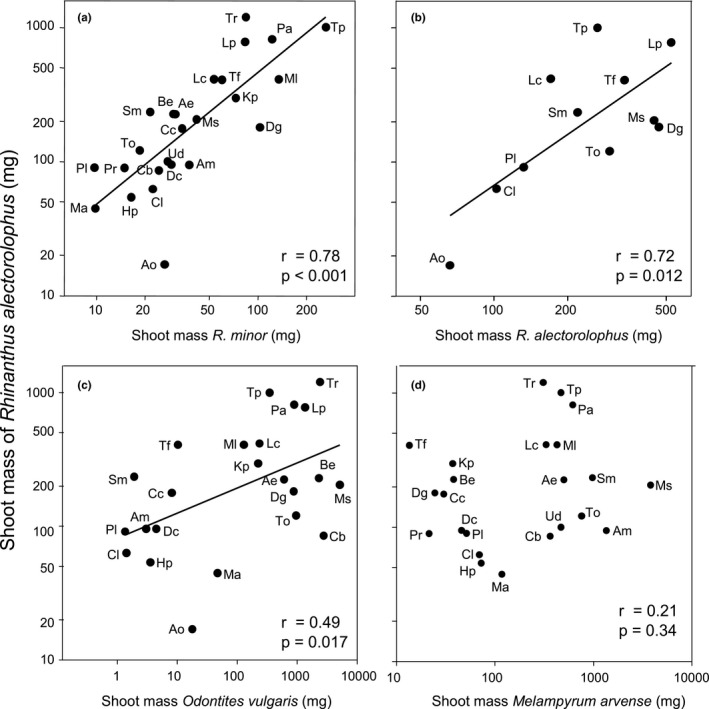
The relationship between the shoot mass of the hemiparasite *Rhinanthus alectorolophus* (this experiment) and that of other hemiparasites grown with the same host plants (a) *R. minor* (this experiment), (b) *R. alectorolophus* (data from Sandner & Matthies, 2018), (c) *Odontites vulgaris* (data from Geppert, 2012), and (d) *Melampyrum arvense*, data from Matthies, 2017)

The biomass of both hemiparasites was strongly influenced by the identity of the host species, and that of *R. alectorolophus* also by nutrient level, but the interaction between the two factors was far from significant (Table 2). The final biomass of both *R. minor* and *R. alectorolophus* varied strongly depending on host species (Figure 2). For *R. minor,* it varied from 9.7 mg when grown with *Plantago* as a host to 263 mg with *Trifolium pratense* (i.e., 27‐fold), and for *R. alectorolophus,* it varied from 17 mg with *Anthoxanthum* to 1,197 mg with *Trifolium repens* (i.e., 70‐fold). The quality of a species as a host for the two species of *Rhinanthus* was strongly correlated (*r* = 0.78, *p* < 0.001; Figure 3a); that is, good hosts for one of the parasites were also good hosts for the other one. The largest deviations from this relationship were observed for *Sanguisorba* and *Trifolium repens* which were better hosts for *R. alectorolophus* than predicted from the performance of *R. minor* with these hosts, and *Anthoxanthum* and *Dactylis* which were poorer hosts than expected. The quality of a certain species as a host for *R. alectorolophus* in the present experiment was also strongly correlated with that of the same species in another experiment (Sandner & Matthies, 2018) with *R. alectorolophus* and several of the same host species (*r* = 0.72, *p* = 0.012; Figure 3b). In contrast, the quality of a species as a host for *R. alectorolophus* and that for the related hemiparasite *Odontites vulgaris* in a previous experiment (Geppert, 2012) with some of the host species was less strongly correlated (*r* = 0.49, *p* = 0.017; Figure 3c), and the correlation with the performance of *Melampyrum arvense* in another experiment (Matthies, 2017) was even weaker (*r* = 0.21, *p* = 0.34; Figure 3d). This was mainly due to a number of grasses that were good hosts for *Rhinanthus*, but not for *Melampyrum* (*Trisetum*, *Dactylis*, *Bromus*, *Cynosurus*, *Koeleria*), and a number of forbs that were good hosts for *Melampyrum*, but rather poor hosts for *Rhinanthus* (*Capsella*, *Urtica*, *Taraxacum, Achillea*).

**TABLE 2:** Analyses of variance of the effect of different host species (*df* = 24) and nutrient level (*df* = 1) on the aboveground biomass, the length of the longest leaf, the height, and the length of the inflorescence of the hemiparasites *Rhinanthus minor* and *R. alectorolophus*. *Df*_res_ = 96 and 160; except for inflorescence length (*df* = 68 and 139). Because the four measures of performance are not independent, the *p*‐values for each effect were adjusted for the false discovery rate (Benjamini & Hochberg, 1995)

Trait	Host		Nutrients		Host × Nutrients	
	*F*	*p*_adj_	*F*	*p*_adj_	*F*	*p*_adj_
*R. minor*						
Biomass (log)	2.6	<0.002	3.4	0.134	1.0	0.464
Leaf length	2.4	0.003	0.4	0.553	1.1	0.464
Height (log)	2.2	0.004	4.1	0.134	1.1	0.464
Inflorescence length (log)	1.8	0.029	0.5	0.553	1.0	0.464
*R. alectorolophus*						
Biomass (log)	6.7	<0.001	16.3	<0.001	0.8	0.933
Leaf length	5.6	<0.001	7.1	0.008	0.6	0.933
Height (log)	3.6	<0.001	7.6	0.008	0.8	0.933
Inflorescence length (log)	5.6	<0.001	9.9	0.004	0.7	0.933

The biomass of *R. alectorolophus* with all host species was much higher than that of *R. minor*, on average by 370%. Higher nutrient levels increased the biomass of *R. alectorolophus* by 87% while the effect on the biomass of *R. minor* (+47%) was not significant (Table 2). The effect of nutrients was similar for parasites grown with different hosts (no significant host × nutrient interaction). The final biomass of the parasites was to a significant extent determined early during development. After four weeks of growth, parasites grown with different hosts already varied strongly in their leaf length (*R. minor: F*_24,121_ = 2.3, *p* < 0.01; *R. alectorolophus: F*_24,185_ = 2.5, *p* < 0.001). Variation in mean leaf length with different hosts at that time could already predict 28% (*R. minor*) and 33% (*R. alectorolophus*) of the variation in final parasite biomass.

A number of traits at harvest were strongly correlated with biomass in both *R. minor* and *R. alectorolophus*: length of the longest leaf (*r* = 0.81 and *r* = 0.87), height (*r* = 0.88 and *r* = 0.92), and total length of the inflorescence of flowering plants, an estimate of reproduction (*r* = 0.87 and *r* = 0.91). They were therefore influenced by the treatments in a similar way as biomass (Table 2). Time until flowering was influenced by the identity of the host species (*F*_24,139_ = 2.1, *p* < 0.01) and nutrient level (*F*_1,139_ = 4.3, *p* < 0.05) in *R. alectorolophus*, but not in *R. minor* (*F*_24,68_ = 0.87, *p* = 0.64 and *F*_1,68_ = 0.50, *p* = 0.48). The mean starting date of flowering varied in *R. alectorolophus* from 46 days after planting with *Lolium* to 59 days with *Daucus* as a host. Time to flowering was determined to a considerable degree by early size (leaf length at 4 weeks). Both in *R. minor* (*r* = −0.32, *p* < 0.001) and in *R. alectorolophus* (*r* = −0.58, *p* < 0.001), the length of time until flowering was shorter for large than for small plants.

### 3.2 Effects of host traits on parasite biomass

In linear mixed models relating the biomass of *R. minor* and *R. alectorolophus* to nutrient level and all the various host traits, only nutrient level (*p* = 0.076 and *p* < 0.001) and the functional group of the hosts (*p* = 0.038 and *p* = 0.003) influenced the performance of the parasites. In contrast, neither the nutrient status of the typical habitat of a host (*p* = 0.61 and *p* = 0.21), nor the mean seed mass of a host (*p* = 0.68 and *p* = 0.87), or the mean biomass of a host at the start of the experiment (*p* = 0.73 and *p* = 0.40), the mean final biomass of a host grown with parasites (*p* = 0.14 and *p* = 0.25) had a significant effect on the performance of the parasites. The mean biomass of a host grown without a parasite, a measure of its growth potential, had also no effect (*p* = 0.90 and *p* = 0.33), nor had the mean RGR of a host (*p* = 0.79 and *p* = 0.40). Similarly, only nutrient level and host functional group were part of the best models with the lowest AICc. The ΔAICc between these best models and the best models including further explanatory variables was 3.0 (*R. minor*) and 5.2 (*R. alectorolophus*).

**FIGURE 4:**
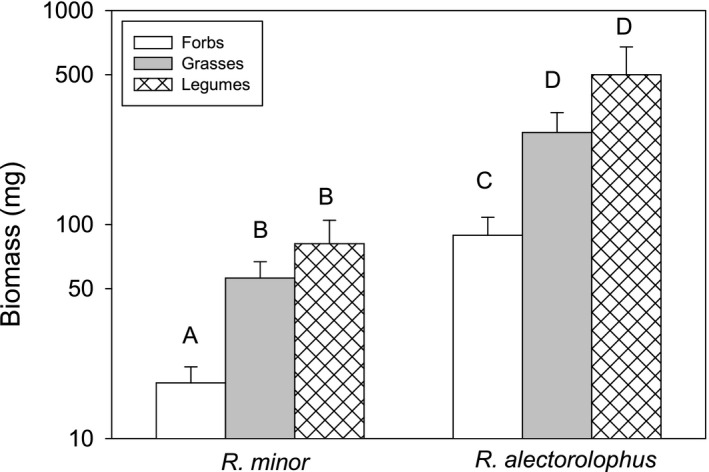
Biomass of the hemiparasites *Rhinanthus minor* and *Rhinanthus alectorolophus* with forbs, grasses, and legumes as hosts. Bars with the same letter within a species are not significantly different at the 0.05 level (Tukey‐test)

In the best models, host quality as measured by the mean biomass of the parasites differed strongly among the three functional groups grasses, legumes, and nonleguminous forbs for both *R. minor* (χ^2^ = 32.6, *p* < 0.001) and *R. alectorolophus* (χ ^2^ = 30.0, *p* < 0.001). The mean biomass of both *R. minor* and *R. alectorolophus* grown with a legume (+347% and +463%; both *p*_adj_ < 0.001) or with a grass (+209% and +202%; *p*_adj_ < 0.001 and *p*_adj_ = 0.002) was much higher than when grown with a nonleguminous forb (Figure 4), while the difference between the performance of parasites grown with a legume and a grass was not significant (*p*_adj_ = 0.48 and *p*_adj_ = 0.21).

At the individual level, the biomass of the hosts grown in the same pot as the parasites had no significant effect on parasite biomass in addition to that of host species and nutrient level, neither for *R. minor* (*F*_1,95_ = 0.39, *p* = 0.54) nor for *R. alectorolophus* plants (*F*_1,159_ = 2.0, *p* = 0.16).

### 3.3 Influence of the parasites on the growth of the host plants and total productivity

Biomass varied among the 25 host species and was higher at high‐nutrient levels (Table 3). However, the host species differed in their response to higher nutrient levels as shown by the host × nutrient interaction. The presence of the parasites reduced the biomass of the host plants, but not very strongly. *R. minor* reduced mean host biomass by 12% and *R. alectorolophus* by 19%. While the mean negative effect of *R. alectorolophus* on the hosts was stronger than that of *R. minor*, the negative effect of the parasites relative to their own size was far stronger for *R. minor*: 1 mg of biomass of *R. minor* caused on average a loss of 4.0 mg of host mass, while 1 mg of biomass of *R. alectorolophus* only caused a loss of 1.3 mg in host mass.

**TABLE 3:** Analyses of variance of the effects of host species, nutrient level, and parasite treatment (no parasite, + *Rhinanthus minor*, + *R. alectorolophus*) on the aboveground biomass of hosts and total aboveground productivity (host + parasite)

Source	*df*	Host biomass		Total biomass	
		*F*	*p*	*F*	*p*
Host species	24	64.4	<0.001	63.8	<0.001
Nutrient level	1	101.9	<0.001	120.5	<0.001
Parasite treatment	2	24.0	<0.001	8.9	<0.001
Host × nutrient level	24	1.6	0.030	1.8	0.009
Host × parasite treatment	48	1.3	0.071	1.9	<0.001
Nutrients × parasite treatment	2	1.8	0.174	1.1	0.328
H × N × P	48	1.0	0.535	1.0	0.412
Residual	456				

**FIGURE 5:**
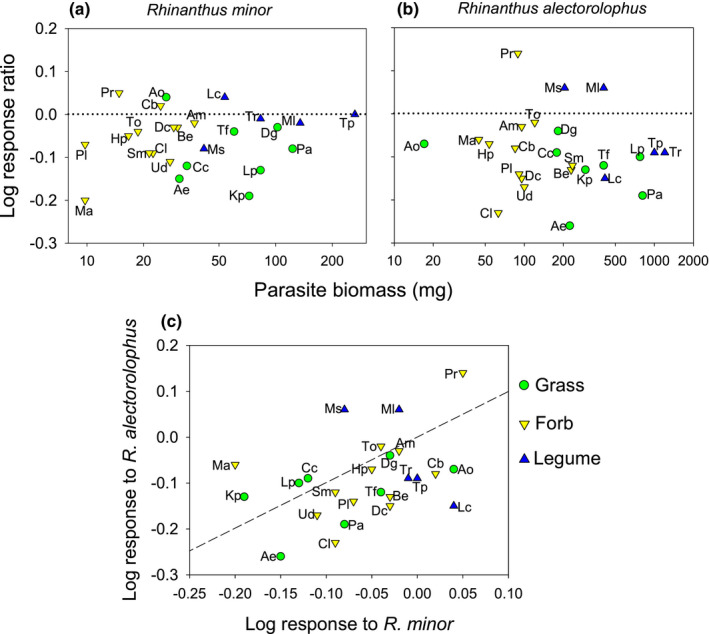
The effect of the hemiparasites *Rhinanthus minor* and *Rhinanthus alectorolophus* on the biomass of the 25 different host species in relation to parasite biomass. Shown is the log‐response ratio = log (mean biomass of host with a parasite/mean biomass of host without the parasite). Response of the biomass of the different hosts to (a) the presence of *R. minor*, and (b) that of *R. alectorolophus*. Negative log‐response ratios indicate that the biomass of a species is reduced by the hemiparasite. (c) The relationship between the response of the host species to *R. minor* and *R. alectorolophus*. Species above the dashed line are more strongly suppressed by *R. minor* than by *R. alectorolophus*, for those below the line the opposite is the case. For abbreviations of host names, see Table 1

The effect of the parasites on host growth did not depend on nutrient level, but there was strong evidence for *R. alectorolophus* and much weaker evidence for *R. minor* that the effect of the two parasites on the hosts varied among species (Table 3, Figure 5). The mean reduction in the biomass of a host species by the two parasites as measured by the log‐response ratio of their biomass to parasite presence was not related to the mean biomass of the parasites (*R. minor*: *r* = 0.13, *p* = 0.53; *R. alectorolophus*: *r* = 0.11, *p* = 0.60), that is, damage to a host was not related to the benefit it provided for the parasites (Figure 5a,b). Some species such as *Papaver, Taraxacum,* and *Dactylis* were not or hardly damaged by either of the parasites and several species proved to be tolerant of parasitism; that is, they were good hosts for the parasites which produced a lot of biomass with them, but suffered relatively little reduction in biomass through parasitism: *Trifolium repens*, *T. pratense*, and *Medicago lupulina* were tolerant of both parasites, while *Dactylis* and *Poa* tolerated parasitism by *R. minor* and *Lolium* was tolerant of *R. alectorolophus*.

A few species were more strongly damaged by *R. minor* (e.g., *Myosotis*, *Medicago sativa,* and *M. lupulina*), but most hosts were more strongly negatively affected by *R. alectorolophus* than by *R. minor* (Figure 5c). However, hosts that were strongly damaged by one of the parasites tended also to be strongly damaged by the other one, as shown by the positive correlation between the log‐response ratios of the effects of the two parasite species on the same hosts (*r* = 0.38, *p* = 0.061). The three functional groups grasses, forbs, and legumes did not vary in the mean degree of biomass reduction due to *R. minor* (*F*_2,22_ = 1.66, *p* = 0.21) and *R. alectorolophus* (*F*_2,22_ = 1.44, *p* = 0.26).

**FIGURE 6:**
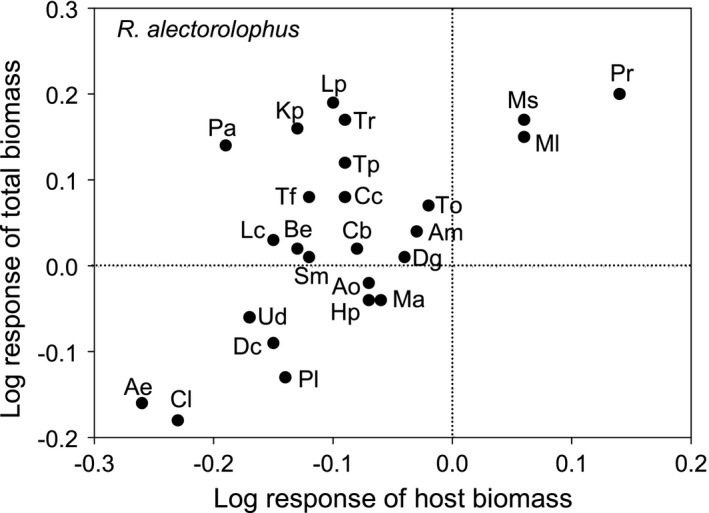
The relationship between the reduction in total aboveground biomass (parasite and host) and the reduction in host biomass for the hemiparasite *Rhinanthus alectorolophus*. Shown are log‐response ratios = log (mean biomass with a parasite/mean biomass without the parasite). Negative log‐response ratios indicate that host biomass or productivity is reduced by the hemiparasite. For abbreviations of host names, see Table 1

Total aboveground productivity per pot (host + parasite biomass) varied depending on the host species (Table 3) and was on average 26% higher at high‐nutrient levels. The effect of the presence of a hemiparasite on productivity also differed between the two parasite species and the hosts, while it was hardly influenced by nutrient level. Separate analyses for the two parasites showed that *R. minor* tended to slightly reduce overall productivity per pot (−5%, *F*_1,296_ = 2.86, *p* = 0.092). This effect did not differ among host species (*F*_24,296_ = 0.88, *p* = 0.63). In contrast, the overall effect of the presence of *R. alectorolophus* on total aboveground productivity was positive (+9%, *F*_1,360_ = 8.71, *p* = 0.003), but differed strongly depending on the host species (*F*_24,360_ = 2.77, *p* < 0.001; Figure 6). Although the parasite *R. alectorolophus* reduced the biomass of nearly all hosts, it nevertheless increased total aboveground biomass per pot with many species, in particular *Poa*, *Koeleria*, *Lolium*, *Trifolium repens,* and *T. pratense*, because its own biomass production more than compensated for the loss of host mass by parasitism (upper left quadrant in Figure 6). However, with other host species such as *Arrhenatherum* or *Chrysanthemum*, the parasite reduced both host mass and total productivity per pot (lower left quadrant in Figure 6).

## 4 DISCUSSION

### 4.1 Influences on the performance of the two hemiparasite species

The performance of the two hemiparasites *R. minor* and *R. alectorolophus* grown with the same host species was closely correlated indicating that at least for two congeneric species of root hemiparasites grown under the same conditions, the relative quality of individual species as hosts is very similar. Moreover, the performance of *R. alectorolophus* in the present experiment was also significantly correlated with that of *R. alectorolophus* in a previous experiment involving many of the same hosts (Sandner & Matthies, 2018) and with that of the related hemiparasite *Odontites vulgaris* in another experiment (Geppert, 2012), although to a lesser degree. Significant correlations between the performance of the hemiparasites were found although the three experiments differed in conditions such as the number of host individuals per pot, soil type, and nutrient levels, which are known to influence hemiparasite–host interactions. In contrast, the performance of *Rhinanthus* was poorly correlated with that of the hemiparasite *Melampyrum arvense* grown with many of the same species (Matthies, 2017). This could be due to different growth conditions in the two experiments, but the strongly different qualities of some species such as *Taraxacum*, *Dactylis,* and *Koeleria* as hosts for *Rhinanthus* and *Melampyrum* suggest that there are also specific interactions between hemiparasite–host pairs. However, some species were consistently poor or good hosts for all four species of hemiparasites (*R. minor*, *R. alectorolophus*, *O. vulgaris*, *M. arvense*), suggesting that they might be generally good or poor hosts. The legumes *Trifolium pratense*, *Medicago sativa,* and *M. lupulina* were good hosts for all four species of hemiparasites, while *Plantago* was a consistently poor host for all the hemiparasites, as were *Myosotis* and *Hieracium*. *Plantago* was also found to be a poor host for *R*. *minor* in previous studies (Barham, 2010; Cameron & Seel, 2007; Rowntree et al., 2014), which has been attributed to its defense reaction to parasite attack (Cameron & Seel, 2007). Nothing is known about possible defense reactions in *Myosotis* and *Hieracium*, but both forbs were hosts of intermediate quality for *Melampyrum arvense* (Matthies, 2017), suggesting that possible defense mechanisms might be specific against certain parasites like *Rhinanthus*.

The influence of host species identity on the performance of the two *Rhinanthus* species was very strong. The mean biomass of *R. minor* varied 27‐fold and that of *R. alectorolophus* 70‐fold depending on the host species. This range in host quality is higher than that observed in other studies of the performance of *Rhinanthus* spp. with different hosts (Hautier et al., 2010; De Hullu, 1984; Rowntree et al., 2014; Seel et al., 1993) and higher than the range observed for other hemiparasites (Calladine et al., 2000; Guo & Luo, 2010; Marvier, 1996; Radomiljac, 1999), but lower than the range found for *Melampyrum arvense* when grown with 25 host species (171‐fold at low nutrients; Matthies, 2017). The large variation in the performance of *Rhinanthus* in the present experiment can be related to the large number of host species used which increased the probability that both very poor and very good hosts were among the species studied. Indeed, the variation in the performance of *Rhinanthus* with different hosts species in previous studies increased with the number and diversity of hosts studied: The biomass of *R. alectorolophus* grown with 9 hosts (only grasses) varied 2.4‐fold (Hautier et al., 2010), that of *R. minor* grown with 9 grasses and forbs 7‐fold (Rowntree et al., 2014), that of *R. minor* grown with 11 species 13‐fold (Seel et al., 1993), that of *R. alectorolophus* with 13 species 11‐fold (Sandner & Matthies, 2018), and that of *R. angustifolius* grown with 18 species 20‐fold (De Hullu, 1984). However, differences in growth conditions and the length of the growth period may also have contributed to the differences among studies.

The quality of a species as a potential host for a root hemiparasite may depend on many aspects of their interactions. Parasites may not attach to the roots of a potential host because it does not stimulate haustoria formation, the haustoria may have problems to penetrate the roots due to the structure or thickness of the roots, and host roots may defend themselves by blocking haustoria (Cameron et al., 2006; Govier, 1966; Yeo, 1964). Moreover, the quantity and quality of compounds provided by a host may vary between species, as grasses have been found to provide mainly carbon while legumes provided nitrogenous compounds (Govier et al., 1967). The ability of host shoots to capture more of the resources taken up by the host roots than the parasite may also vary among species, as well as the impact of the host on hemiparasite growth by shading (Matthies, 1995a). The variation in growth of the two species of *Rhinanthus* with the different host species could not be explained by several characteristics of the host species that were found in other experiments to have an influence, like their size when grown with or without a parasite, their growth rate or realized niche with respect to nutrients (Hautier et al., 2010; Marvier, 1996; Matthies, 2017). Thus, large hosts did not provide more solutes to the parasites than small ones and fast‐growing species were not better hosts than slow‐growing ones.

In contrast, there were significant differences among functional groups in their quality as host plants. Of the 25 species studied, the five legumes and nine grasses were on average much better hosts for both *R. minor* and *R*. *alectorolophus* than the eleven nonleguminous forbs. The often observed large benefits of legume hosts for hemiparasites (Lu et al., 2014; Radomiljac, 1999; Rowntree et al., 2014; Seel et al., 1993; Tennakoon & Pate, 1996; Yeo, 1964) could be attributed to their symbiosis with nitrogen‐fixing bacteria and consequent high supply of nitrogen (Govier et al., 1967, Jiang et al., 2008). Similarly, a strong preference for potentially N‐fixing species as hosts (legumes and nonlegumes) was found for the hemiparasite *Santalum acuminatum* (Tennakoon, Pate & Arthur, 1997). The suitability of grasses has been attributed to the weak defense of their roots against parasite attack (Cameron et al., 2006; Rümer et al., 2007). However, these general effects masked considerable variation within the functional groups. The performance of the *Rhinanthus* with forbs as hosts was poor, but this was also the case with several of the grasses such as *Anthoxanthum*, *Bromus*, *Arrhenatherum,* and *Cynosurus*. In a comparison of nine host species, Rowntree et al. (2014) also found forbs to be the least beneficial hosts for *R. minor*. In contrast, in a study of the growth of *R. alectorolophus* with 13 host species (Sandner & Matthies, 2018), grasses were the best and legumes the worst hosts. However, this was due to the presence of two legumes (*Anthyllis* and *Onobrychis*) in the experiment that were very poor hosts. Thus, while most legumes generally appear to be good and forbs rather poor hosts for *Rhinanthus* as concluded by Cameron and Phoenix (2013), individual species may deviate from this general pattern. Why some legumes were poor hosts in studies of hemiparasite–host interactions is usually not clear, but Govier (1966) found that *Trifolium incarnatum* blocked the haustorium of the hemiparasite *Odontites verna* by producing a layer of a substance (probably tannin) between the appressorial cells of the haustorium and the stele of the clover.

The identity of the host species influenced also traits of the parasites other than biomass such as their leaf length, height, inflorescence length, and time until flowering, but this was to a large degree an effect of the effect of host identity on parasite size. Parasites attached to good hosts grew faster and started to flower earlier. Pollinations early in the season may thus occur mainly between parasite individuals that have been successful in parasitizing certain host species that are most beneficial. As the ability to successfully exploit individual host species has a genetic component (Ahonen et al., 2006; Rowntree, 2014; Sandner & Matthies, 2017), this would result in assortative mating and might facilitate the evolution of genotypes adapted to specific hosts. However, there is yet little evidence for the evolution of specialization on hosts in *Rhinanthus* (Ahonen et al., 2006; Mutikainen et al., 2000).

Higher nutrient levels did not affect the survival and increased the growth of both species of hemiparasite, although in the case of *R. minor,* this effect was not significant. Nutrient levels in the current study were thus not sufficient to change the balance in the competition for light between the hemiparasites and their hosts in favor of the host plants which would have resulted in increased mortality of young hemiparasites (Matthies, 1995; Matthies & Egli, 1999; Mudrák and Lepš, 2010; Těšitel, Těšitelová, et al., 2015). Because the host plants are for hemiparasites simultaneously beneficial sources of water, nutrients, and carbon, but also competitors for light, hemiparasites are restricted to habitats of low‐nutrient availability (Matthies, 1995; Těšitel, Fibich, et al., 2015). However, in experimental studies using pots negative effects of high‐nutrient levels on hemiparasites are not always observed (Borowicz & Armstrong, 2012; Korell et al., 2020; Matthies & Egli, 1999) and will depend on maximum nutrient levels, host density, and host age.

### 4.2 Effects of the two hemiparasite species on the hosts

The negative effects of root hemiparasites on the growth of their hosts are often very strong (Hautier et al., 2010; Korell et al., 2020; Matthies, 2017; Matthies & Egli, 1999; but see Tennakoon, Pate & Fineran, 1997). In a meta‐analysis, Ameloot et al. (2005) concluded that *Rhinanthus* spp. on average reduced host biomass by 60% in pot and 40% in field experiments. In later experiments, *R. alectorolophus* reduced the mass of its host species by between 9% and 37% (Sandner & Matthies, 2018), and by 56% (Korell et al., 2020), and *R. minor* by 26% (Bardgett et al., 2006). In the current study, host damage was far less severe. The mean reduction of host biomass was 12% by *R. minor* and 19% by *R. alectorolophus*, and even the biomass of the most strongly affected hosts was only reduced by 36% (*R*. *minor*) and 45% (*R. alectorolophus*). The lower impact of the parasites on their hosts could be due to the fact that three host individuals were planted with each parasite per pot, thus potentially reducing the effect of the parasite (but see Korell et al., 2020). Moreover, the plants were well watered and the negative effects of hemiparasites on their hosts may be strongest if either water or nutrients are strongly limiting plant growth, as water and nutrients are the two key resources that root hemiparasites extract (Těšitel, Těšitelová, et al., 2015).

The negative impact of the parasites on the growth of the hosts differed strongly among host species, but the effect of the two *Rhinanthus* species was correlated, although there was a lot of variation, indicating that the two parasite species made similar relative demands on the hosts. The fact that a host species suffers no or little damage by a parasite can be due to constitutive resistance of the root system of a host to parasitic attack or to a successful defense reaction (Atsatt, 1983; Cameron & Seel, 2007). This is the likely explanation for the low damage in species that provided little benefit to the parasites such as *Hieracium* and *Papaver*. However, several legumes (*Medicago lupulina*, *M. sativa*, *Trifolium pratense*, *T. repens*, *Lotus*) and the grass *Dactylis* supported large parasites of one or both *Rhinanthus* species and were thus good hosts, but were hardly suppressed by the parasites. These species were thus tolerant of parasitism. Tolerance against parasitism in *Lotus* and *Trifolium pratense* has also been observed in another experiment with *R. alectorolophus* (Sandner & Matthies, 2018), and tolerance against the related hemiparasite *Melampyrum arvense* was found for several of the same legume species as in the current experiment (*Lotus*, *Trifolium pratense*, *T. repens;* Matthies, 2017). All these species grew vigorously and produced a lot of biomass, and vigorous growth is also a typical constitutive trait of plants that are tolerant of the attack of herbivorous insects (Fornoni, 2011). Tolerance of the legumes may have been facilitated by their mutualistic symbiosis with nitrogen‐fixing *Rhizobia*. The fact that several host species were tolerant of parasitism could partly explain the low correlation between the benefit a parasite derived from a certain host species and the damage in terms of reduced biomass it caused the host. This weak relationship indicates that the damage to the host cannot be explained simply by the amount of resources extracted by the hemiparasites. In contrast, in *Melampyrum arvense* the benefit of a host species for the parasite and the damage to this host have been found to be strongly correlated (Matthies, 2017).

It has been suggested that the negative impact of hemiparasites on their hosts will be particularly strong at low levels of nutrient availability (Matthies, 1995a; Těšitel, Těšitelová, et al., 2015), because under high‐nutrient conditions the loss of nutrients to the parasite will be less detrimental to the host plants. Some studies have found support for this notion (Liu et al., 2017; Matthies & Egli, 1999; Těšitel, Těšitelová, et al., 2015), but others found no influence of nutrient level on host damage by hemiparasites (Bardgett et al., 2006; Korell et al., 2020; Mudrák and Lepš, 2010) or only very small effects (Matthies, 2017). Similarly, no increased damage to the hosts at low‐nutrient levels was observed in the present study. These conflicting results indicate that variation in other factors strongly influences the effect of nutrients on hemiparasite–host interactions.

### 4.3 Effects on total productivity

Because hemiparasites have a lower resource use efficiency than their hosts and affect host photosynthesis, negative effects of the presence of hemiparasites on total productivity (host and parasite combined) can be expected (Hautier et al., 2010; Matthies, 1995). To obtain nutrients from the roots of their host plants, hemiparasites have very high rates of transpiration, even in the dark when autotrophic plants close their stomata (Lechowski, 1996; Press et al., 1988), and while parasites may accumulate very high concentrations of nutrients in their tissues (Pate et al., 1990), their own rates of photosynthesis are similar to or lower than those of their hosts (Lechowski, 1996; Press et al., 1993). Hautier et al. (2010) even predicted based on a model that the presence of hemiparasites will always reduce total productivity. However, the results of empirical studies on the effect of hemiparasites on total productivity are inconsistent. Some studies have found a reduction of overall productivity due to the presence of a hemiparasite (Hautier et al., 2010; Korell et al., 2020; Matthies, 1995a, 1995b; Matthies, 1996; Matthies & Egli, 1999; Mudrák & Lepš, 2010), while others have found an increase of productivity with at least some hosts (Matthies, 2017; Sandner & Matthies, 2018) or host combinations (Joshi et al., 2000; Sandner & Matthies, 2018).

In the present experiment, the two species of *Rhinanthus* had different effects on productivity. Total aboveground productivity was on average reduced by *R. minor*, but actually increased by *R. alectorolophus,* indicating that in many parasite–host combinations the loss in host biomass due to parasitism was more than compensated by the carbon gain through the photosynthesis of *R. alectorolophus*. However, because root hemiparasites invest very little biomass into their own roots and instead rely on the resources taken up by the roots of their hosts (Matthies, 1995, 2017), the negative effect of the parasites on total productivity (above ground and below ground) could have been stronger.

## 5 CONCLUSIONS

The performance of the two hemiparasites *R. minor* and *R. alectorolophus* grown with the same host species was strongly correlated, and also correlated with that of *R. alectorolophus* and *Odontites* in other experiments, in spite of differing conditions. Moreover, the two *Rhinanthus* species had related effects on the host species. This shows that the interactions between closely related root hemiparasites and individual host species are similar and that certain root traits and defense mechanisms of potential hosts may be effective against several hemiparasites. However, the weak correlation between the performance of *Rhinanthus* and *Melampyrum* with the same hosts shows that there are also specific interactions between pairs of hemiparasite–host species. The differences in the response of *Rhinanthus* and *Odontites* versus that of *Melampyrum* could be related to their phylogenetic relationships, as the genus *Odontites* is more closely related to *Rhinanthus* than is *Melampyrum*, which forms a sister group to other Orobanchaceae (Těšitel, Říha, et al., 2010).

The results of this study confirmed that legumes are in general, although not universally, very good hosts for hemiparasites and that some of them are tolerant of parasitism (Matthies, 2017; Sandner & Matthies, 2018). Both phenomena can be related to their symbiosis with nitrogen‐fixing *Rhizobia*, because the high nitrogen content of legumes benefits the parasites, while legumes will also be more capable to compensate for the loss of nitrogen to the parasites than plants from other functional groups (Matthies, 2017).

Both *R. minor* and *R. alectorolophus* have been used as model species for the study of host–hemiparasite relationships. However, the present study revealed important differences between the two parasites. The biomass of *R. alectorolophus* was much higher than that of *R. minor* with every host species and *R. alectorolophus* also reacted more strongly with increased growth to nutrient addition than did *R. minor*, indicating that *R. alectorolophus* is the more competitive species in fertile habitats. The results are in line with the different habitats of the two parasites. While *R. minor* is a typical species of nutrient‐poor grasslands, *R. alectorolophus* grows in more mesotrophic grasslands and also formerly occurred as an agricultural weed (Ellenberg et al., 1992; Hartl, 1974). The fact that *R. alectorolophus* in contrast to *R. minor* increased overall productivity and caused less damage to the hosts in relation to its own biomass shows that *R. alectorolophus* has a greater capacity to use nutrients obtained from the host to increase its own photosynthesis, makes less demands on its hosts, and is thus less parasitic than *R. minor*. In line with this, carbon taken up from the host accounted for 50% of total carbon in *R. minor* (Těšitel, Plavcová, et al., 2010) but only for 10%–40% in *R. alectorolophus* (Těšitel, Těšitelová, et al., 2015), depending on host and growth conditions.

## CONFLICT OF INTEREST

None declared.

## AUTHOR CONTRIBUTION

**Diethart Matthies**: Conceptualization (lead), data curation (lead), formal analysis (lead), investigation (lead), methodology (lead), project administration (lead), resources (lead), validation (lead), visualization (lead), writing – original draft (lead), and writing – review and editing (lead).

## Data Availability

Data from this study are available from the Dryad Digital Repository: https://doi.org/10.5061/dryad.n5tb2rbw8.
